# Thermomechanical Devulcanisation of Ethylene Propylene Diene Monomer (EPDM) Rubber and Its Subsequent Reintegration into Virgin Rubber

**DOI:** 10.3390/polym13071116

**Published:** 2021-04-01

**Authors:** Dávid Zoltán Pirityi, Kornél Pölöskei

**Affiliations:** Department of Polymer Engineering, Faculty of Mechanical Engineering, Budapest University of Technology and Economics, Műegyetem Rakpart 3., H-1111 Budapest, Hungary; pirityid@pt.bme.hu

**Keywords:** elastomers, devulcanisation, rubber recycling, two-roll mill, thermomechanical treatment

## Abstract

Rubber waste remains a challenge for material science because its covalently cross-linked structure hinders the establishment of the circular economy of rubber. Devulcanisation may provide a solution, as it converts rubber vulcanisates back into their original, uncured state. Devulcanised rubber may be revulcanised or incorporated into virgin rubber, thus waste is utilized and the use of primary resources is reduced at the same time. In this paper, we treated sulphur-cured EPDM (ethylene propylene diene monomer) rubber on a two-roll mill at various temperatures and frictions. We determined the effectiveness of devulcanisation via Horikx’s analysis, which suggested that low devulcanisation temperatures would result in a 50% decrease in cross-link density with minimal polymer degradation. The devulcanisate was recycled via two methods: (a) revulcanisation with extra curing agents, and (b) mixing it with various amounts of the original rubber mixture, preparing rubber samples with 25, 50, 75, and 100 wt% recycled content. Tensile tests revealed that the samples’ elastic properties were severely compromised at 75 and 100 wt% devulcanisate contents. However, tensile strength decreased only by 15% and 20% for revulcanisates containing 25% and 50% recycled rubber, respectively.

## 1. Introduction

Rubber waste management is one of the challenges of materials science that has yet to be solved. With an estimated market of 30 million tons per year [1,2,3], the rubber industry is a major producer of waste. Rubber products do not break down, nor do they melt, so conventional reprocessing techniques are not suitable for rubber recycling [4]. Current recycling approaches are still similar to those developed in the 1850s, when Charles Goodyear first patented the addition of ground rubber waste into virgin rubber mixtures, and Hall patented the thermal plasticization of rubber shoe soles [5]. The lack of true innovation in rubber recycling in the past 170 years has led to the current practice of incinerating or downcycling rubber waste [5]. Devulcanisation has been proposed as a viable solution for this problem, as it would ideally convert rubber waste back into its original, uncured form. During this process, cross-links are selectively broken down, while the polymer chains retain their original length. In practice, true devulcanisation has never been achieved due to undesired chain degradation and oxidation. Ultimately, the side reactions compromise the mechanical properties of recycled rubber [6].

EPDM (ethylene propylene diene monomer) rubber is mainly vulcanized by two methods: (a) with sulphur, creating sulphuric cross-links; or (b) with peroxides, linking polymer chains through C–C covalent bonds. Two main factors are generally mentioned in connection with devulcanisation. On the one hand, the bond energy of sulphuric cross-links (C–S and S–S covalent bonds) is significantly lower than that of C–C covalent bonds comprising the polymer backbone [7]. Consequently, when a rubber vulcanisate is heated, sulphuric cross-links are more likely to break than bonds in the polymer chain. Still, considering the ratio of cross-links to other bonds in an elastomer, a large number of C–C bonds will break when exposed to heat only. On the other hand (according to Fukumori et al. [8,9]), the elastic constant of C–C bonds is about 30 times higher than that of S–S bonds, which means that high shearing forces would cause sulphuric cross-links to stretch to a greater extent than other bonds, making them more susceptible to cleavage. Both approaches imply that peroxide-cured rubbers cannot be effectively devulcanised, as their cross-links are made of the same covalent bonds as their polymer backbone. Thermomechanical stimuli would lead to the random degradation of peroxide-cured rubber, but they might be effective in the selective breakdown of sulphuric cross-links [10].

Several devulcanisation strategies have been proposed and investigated in the academic literature. Microbiological treatment involves the use of microorganisms that consume the sulphuric components of organic materials to obtain energy [11,12]. Microwave devulcanisation harnesses the fact that microwaves can heat matter uniformly with little surface effect [4,13,14,15]. Thermomechanical devulcanisation ranges from extruders [16] and high shear mixers [17] to conventional rubber processing technologies (mills and internal mixers) [18,19]. During ultrasonic devulcanisation, ultrasound waves are used to break down rubber networks; this technology is often coupled with an extruder, combining the effects of thermomechanical and ultrasonic treatments [20,21]. Chemical devulcanisation is also widely studied, but its industrial significance is limited due to the need for solvent regeneration [22,23]. Currently, only the thermomechanical and microwave methods have high enough productivity for industrial use. Thermomechanical and ultrasonic devulcanisation technologies are preferable, due to their better scalability and higher selectivity for cross-link scission [6,24].

Sutanto et al. [7] and Macsiniuc et al. [25] devulcanised EPDM rubber in a batch internal mixer and achieved high degrees of devulcanisation (up to 75%). In general, temperature, shearing rate, fill ratio, and residence time were the most important factors impacting devulcanisation. The combination of high temperature and shearing rate often results in severe chain degradation, while at inadequate shearing rates, devulcanisation may not occur at all.

One of the most widespread evaluation techniques to determine the effectiveness of devulcanisation was developed by Horikx in the 1950s [26]. His method enables us to determine whether a polymer degradation is dominated by the selective breakdown of cross-links (devulcanisation), or chain degradation. He identified two distinct theoretical scenarios based on the Flory–Huggins theory [27,28]. The first scenario assumes that the polymer was degraded via a statistically random distribution of chain scission events. Equation (1) correlates the sol fraction and the cross-link density of the degraded polymer:

(1) $$1-\frac{v_{f}}{v_{i}}=1-\frac{{\left(1-\sqrt{s_{f}}\right)}^{2}}{{\left(1-\sqrt{s_{i}}\right)}^{2}}$$

where *ν_i_* [mol/cm^3^] stands for the initial cross-link density, *ν_f_* [mol/cm^3^] stands for cross-link density after degradation, *s_i_* [–] stands for the initial sol fraction of the polymer, and *s_f_* [–] stands for the post-treatment sol fraction of the polymer [10,26].

In the second scenario, it is assumed that the degradation process consists solely of the selective breakdown of cross-links, strictly corresponding with an ideal devulcanisation process. He devised Equation (2) with the same principle of expressing the polymer’s sol fraction as a function of its cross-link density:

(2) $$1-\frac{v_{f}}{v_{i}}=1-\frac{\gamma_{f}{\left(1-\sqrt{s_{f}}\right)}^{2}}{\gamma_{i}{\left(1-\sqrt{s_{i}}\right)}^{2}}$$

where *γ_i_* [–] and *γ_f_* [–] stand for the initial and final cross-linking indices, respectively [10]. The cross-linking index represents the average number of cross-links per polymer chain and can be determined according to Equation (3):

(3) $$\gamma_{x}=v_{x}\frac{M_{n}}{\rho }$$

where *γ_x_* [–] is the cross-linking index, *ν_x_* [mol/cm^3^] is the cross-link density, *M_n_* [g/mol] stands for the number-average molecular weight of the polymer, and *ρ* [g/cm^3^] is the density of the polymer [26]. In this paper, the initial cross-linking index is estimated based on Equation (4) [29]:

(4) $$s_{i}=\frac{\left(2+\gamma_{i}\right)-\sqrt{\gamma_{i}^{2}+4\gamma_{i}}}{2\gamma_{i}}$$

Devulcanisation on its own is not adequate; the application of devulcanisates is also a crucial step in rubber recycling. Three strategies can be distinguished, according to Forrest [30] and Isayev [6]. Under certain conditions, it is possible to produce thermoplastic elastomers by adding devulcanisates or ground waste rubber to conventional thermoplastic polymers [31,32,33]. However, the circular economy of rubber is only achieved when the waste material is used in new rubber vulcanisates. Consequently, adding devulcanisates to virgin rubber mixtures can produce new rubber with some recycled content [34,35]. Revulcanising previously devulcanised rubber can produce 100% recycled rubber [16,36,37].

Jacob et al. [38] mixed ground EPDM vulcanisates into virgin rubber compounds. Their direct recycling method revealed that waste rubber would enhance the mechanical properties of the otherwise unfilled EPDM rubber, thanks to its carbon black content.

Seghar et al. [39] followed similar principles and prepared revulcanised rubber and vulcanisates with various devulcanisate content. They achieved a 90% decrease in cross-link density, with a significant extent of degradative side reactions. Their revulcanisates with up to 10 wt% recycled content had no significant change in mechanical properties compared to virgin rubber. However, a 20 wt% recycled content resulted in a 20% decrease in the samples’ modulus and strength.

Yun et al. [21] achieved an up to 85% decrease in cross-link density for unfilled EPDM via ultrasonic devulcanisation. However, even a 15 phr carbon black content would decrease the degree of devulcanisation to 70%. Additionally, they showed that the carbon black content would shift the devulcanisation process towards the random chain scission curve according to Horikx’s theory. They added the devulcanisates to virgin rubber compounds and prepared vulcanisates at various recycled contents. They revealed that the devulcanisate increased the Young’s modulus of the revulcanisates. The tensile strength and elongation at break of the revulcanisates decreased by around 35%, 45%, 50%, and 70% at 25 wt%, 50 wt%, 75 wt%, and 100 wt% recycled contents, respectively, compared to virgin rubber.

In this paper, we investigated the potential of batch devulcanisation of EPDM rubber on a two-roll mill by treating rubber samples at various temperatures and roll speeds. The use of traditional rubber processing equipment may encourage manufacturers to adopt this recycling technology. We identified the optimal devulcanisation parameters on the tested range using Horikx’s analysis. Subsequently, we revulcanised our devulcanisates and mixed the devulcanisates into virgin rubber mixtures of identical composition at various ratios. Finally, we tested the mechanical and morphological properties of the resulting, partially recycled rubbers.

## 2. Materials and Methods

### 2.1. Materials

Table 1 shows the composition of the EPDM-based rubber mixture used in the experiments. The base polymer, Dutral TER 4047, has an intrinsic Mooney viscosity of 55 at 125 °C and contains 54 wt% ethylene, 41.5 wt% propylene, and 4.5 wt% ethylidene norbornene. The rubber mixture was prepared and provided by Palotás Mix Kft. (Kemeneshőgyész, Hungary), and contained large amounts of fillers: 85 phr of carbon black and 30 phr of dolomite. The rubber composition was created to mimic EPDM rubbers of general use: profiles, gaskets, etc.

Toluene was used as the general solvent for cross-link density and sol content measurements. It was supplied by Fisher Scientific UK (Loughborough, UK).

For the revulcanisation experiments, powdered zinc oxide was supplied by Werco Metal (Zlatna, Romania). Zinc stearate, TBTD, ZDBC, and sulphur were supplied by Ningbo Actmix Rubber Chemicals Co., Ltd. (Ningbo, China).

### 2.2. Preparation of Rubber Vulcanisates

We used a Collin Teach-Line Platen Press 200E (Dr. Collin GmbH, Ebersberg, Germany) hot press to cure the EPDM rubber. Rubber sheets with a 2 mm thickness were obtained. The plates of the press were heated up to 180 °C, and the ultimate pressure was 2.8 MPa. We determined the optimal curing time (t_90_, at which 90% curing is achieved) from rheometer measurements. We applied the pressure gradually to allow for degassing.

**Figure 1:**
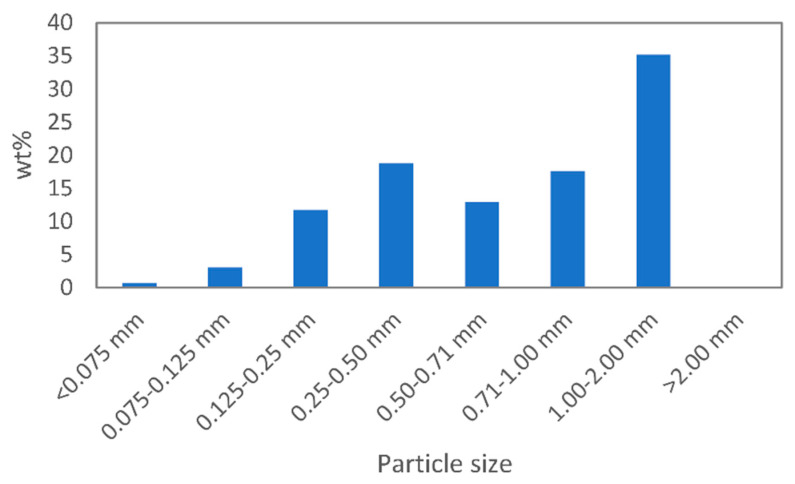
Particle size distribution of the ground EPDM rubber.

We used a Retsch ZM200 (Retsch GmbH, Haan, Germany) cryogenic mill to pulverise the cured rubber sheets. Initially, we cut the sheets into 10 mm × 10 mm pieces and cooled them in liquid nitrogen. The frozen rubber pieces were fed to the pre-cooled milling chamber one by one. The rotor speed was set to 12,000 rpm. The resulting particle size distribution of the ground EPDM rubber is shown in Figure 1.

### 2.3. Devulcanisation

We devulcanised EPDM rubber on a Labtech LRM-SC-11/3E (Labtech Engineering Co. Ltd., Samutprakarn, Thailand) two-roll mill. We fed the material between the rolls in batches of 50 g and allowed the crumbs to pass through the gap 20 times. The rubber powder was allowed to cool between cycles to avoid heat buildup during the process. The temperature and roll speeds were varied according to Table 2. We observed no deviation in the roll temperatures from the set values.

### 2.4. Revulcanisation

dev_80-1.33 was selected as the devulcanisate with the best properties, based on Horikx’s analysis, and hence it was used for revulcanisation experiments. We investigated the revulcanisability of dev_80-1.33 in two ways: (a) on its own, and (b) by adding the original curing system to the devulcanisate. We added the curing agents to the devulcanisate in a Brabender Plasti-Corder (Brabender Technologie GmbH & Co., Duisburg, Germany) internal mixer at a fill ratio of 70% in a 50 cm^3^ mixing chamber at 70 °C for 20 min. The ratio at which the curing system was added to the devulcanisate is shown in Table 3. The devulcanisate that contained additional curing agents was named dev_80-1.33_mix.

We prepared mixtures of the virgin EPDM rubber and dev_80-1.33_mix in the previously mentioned Brabender internal mixer with the same operating conditions. These mixtures, along with a reference virgin rubber and Sample 1, were tested in the rheometer and vulcanised in the compression mould to t_90_ the same way the original rubber vulcanisate had been prepared. Table 4 shows the composition of all revulcanisates.

### 2.5. Testing

We used a MonTech Monsanto R100S rheometer (MonTech Werkstoffprüfmaschinen GmbH, Buchen, Germany) to determine the curing properties of each rubber mixture. We ran the rheometer in isothermal (T = 180 °C) time sweep mode (1.667 Hz, 1° amplitude) for 30 min.

We measured the sol content of the rubber samples via Soxhlet extraction. We placed 10 g of rubber powder into cellulose thimbles and sealed them with cotton wool. We added 200 mL of toluene to the bottom distillation flask and ran the experiments for 20 h, throughout which the top column of the equipment was cooled with tap water. After the extraction, we dried the specimens at 120 °C for 6 h to remove the solvent.

Equation (5) was used to compute the sol content:

(5) $$s=1-\left(\frac{M_{f}}{M_{i}}\right)$$

where *s* [–] stands for sol content, and *M_i_* [g] and *M_f_* [g] stand for the mass of rubber before and after the extraction, respectively.

The sol content obtained from Equation (5) stands for the rubber mixture’s overall sol content. However, Horikx’s theory uses the sol content of the polymer part only. It was safe to assume that the oil, lubricant, and PEG components would be completely extracted from the mixture. Consequently, we devised Equation (6) to solve this issue:

(6) $$s_{corr}=2.431\left(s-\frac{19}{243.1}\right)$$

We measured the cross-link density of the rubber samples according to the ASTM D6814 standard. We immersed the rubber samples in toluene for 72 h (until equilibrium swelling was achieved). We dabbed the swollen rubber powder dry with paper towels and weighed them. We then dried the samples at 120 °C for 6 h and weighed the samples again. Cross-link densities were evaluated using the Flory–Rehner Equation (7):

(7) $$\nu_{x}=-\frac{\left[\ln\left(1-v_{r}\right)+v_{r}+\chi v_{r}^{2}\right]}{\left[V_{1}\left(v_{r}^{1/3}-v_{r}\right)/2\right]}$$

where *ν_x_* [mol/cm^3^] denotes the cross-link density, *χ* [–] means the polymer–solvent interaction parameter (which equals 0.496 for an EPDM-toluene system), *V*_1_ [cm^3^/mol] is the molar volume of the solvent (106.13 cm^3^/mol for toluene), and *v_r_* [–] stands for the volume fraction of rubber in the toluene-swollen rubber sample [10,25]. We applied Equation (8) to estimate *v_r_* from our experimental data, as suggested in the ASTM D6814-02 (2018) standard:

(8) $$v_{r}=\frac{\frac{m_{r}}{\rho_{r}}}{\frac{m_{r}}{\rho_{r}}+\frac{m_{s}}{\rho_{s}}}$$

where *m_r_* [g] stands for the mass of the dried rubber sample, *m_s_* [g] denotes the mass of the solvent absorbed by the swollen rubber (the difference between the masses of the swollen and dry rubber samples), *ρ_r_* [g/cm^3^] is the density of the rubber samples (measured to be 1.23 g/cm^3^), and *ρ_s_* [g/cm^3^] means the density of the solvent (0.867 g/cm^3^ for toluene) [10,25].

We used a ball press to cut tensile and tear strength test specimens out of the compression moulded sheets. We performed tensile tests on a Zwick Z020 (ZwickRoell GmbH, Ulm, Germany) tensile tester with a 20 kN load cell, according to ISO 37:2017. The clamping distance was 60 mm, and the crosshead speed was 500 mm/min. We used the same tensile tester for tear strength tests with a clamping distance of 56 mm and a crosshead speed of 500 mm/min, according to ISO 34-1:2015.

The Shore A hardness of the rubber sheets was determined with a Zwick H04.3150.000 hardness tester, according to ISO 48-4:2018.

We took scanning electron micrographs (SEM) of the fracture surfaces of the tensile test specimens. Surfaces were first sputter-coated with gold and then placed into a Jeol JSM-6380LA (Jeol Lt., Tokyo, Japan) microscope.

## 3. Results and Discussion

### 3.1. Devulcanisation of EPDM Rubber

We fed ground EPDM rubber in batches of 50 g between the rolls of a two-roll mill at nine different operating settings. Afterwards, we measured the sol content and the cross-link density of each devulcanised sample. Table 5 shows the degree of devulcanisation and sol content for all devulcanisates. All values were relatively close to each other, especially considering the standard deviation of the experimental data. The devulcanisation at our testing range resulted in an approximately 50% decrease in cross-link density, while the sol content of the devulcanisates were as low as 3–5 wt%. We achieved the highest degree of devulcanisation at 80 °C with a 1.33 and a 1.67 friction. We found that 100 °C was the least favourable temperature setting, yielding the lowest degree of devulcanisation. The degree of devulcanisation at 120 °C was significantly higher than that at 100 °C, and the results closely resemble the ones obtained at 80 °C. It can be argued that, due to the intrinsic viscosity of rubber at various temperatures, the same friction had different shearing effects. While at 80 °C, devulcanisation was mostly controlled by mechanical forces, it was dominated by thermal effects at 120 °C. This is a possible explanation of why 100 °C was the least effective temperature. It can also justify why friction had little effect at 120 °C, while it affected the degree of devulcanisation and sol content more significantly at 80 and 100 °C. The large friction may have caused slippage at 80 °C; EPDM rubber did not soften at that temperature, making it less likely to stick to the steel rolls, hence the low degree of devulcanisation of dev_80-2.00.

**Figure 2:**
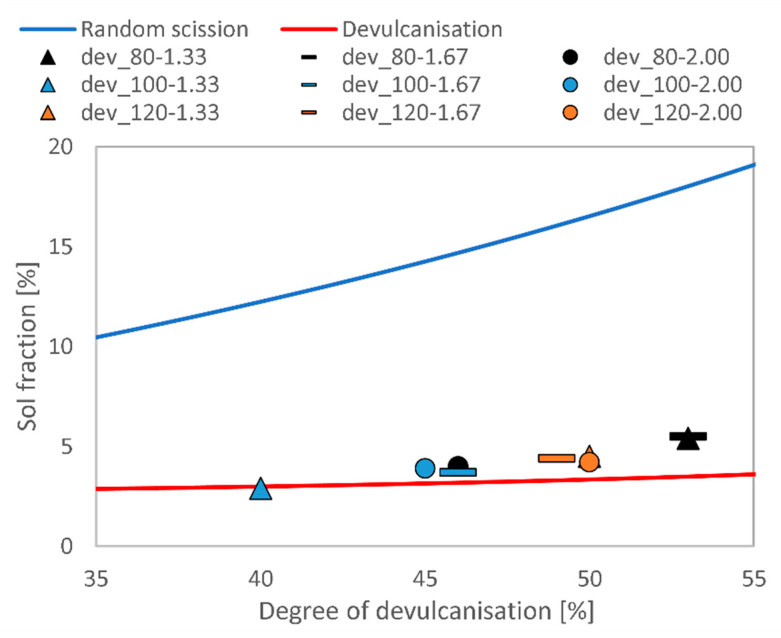
Horikx’s plot for the devulcanisates.

We plotted the above results in Horikx’s plot in Figure 2. The sol fraction is shown on the *y*-axis, while the relative decrease in cross-link density is shown on the *x*-axis. The nine data points are in the vicinity of the theoretical curve that represents devulcanisation. Ultimately, it means that thermomechanical devulcanisation on a two-roll mill at moderate operating conditions decreases the cross-link density of EPDM rubber by around 50%, while the sol content of the rubber stays low. This combination of properties means that polymer molecules were mostly kept intact. When comparing these results to those reported in the scientific literature, we can infer that devulcanisation can work even at low processing temperatures [21]. However, to achieve a larger decrease in cross-link density, the temperature should also be increased. At higher temperatures, polymer degradation is usually more prominent than in our case. Another approach for achieving larger degrees of devulcanisation would be selecting technologies with larger shearing effects. Ultimately, the mild operating conditions we used can offer a good compromise between the degree of devulcanisation and the extent of polymer degradation. It should also be noted that lower operating temperatures correspond to lower energy consumption, so it can be considered a more environmentally friendly recycling approach in the case that the applicability of the devulcanisates is not hindered. According to Yun et al.’s [21] results, carbon black makes the devulcanisation of EPDM rubber less effective. Even though they managed to achieve an 85% degree of devulcanisation with unfilled rubber, they only reached 60% at 60 phr carbon black content. Additionally, the polymer chains degraded severely in the latter case.

### 3.2. Revulcanisation

**Figure 3:**
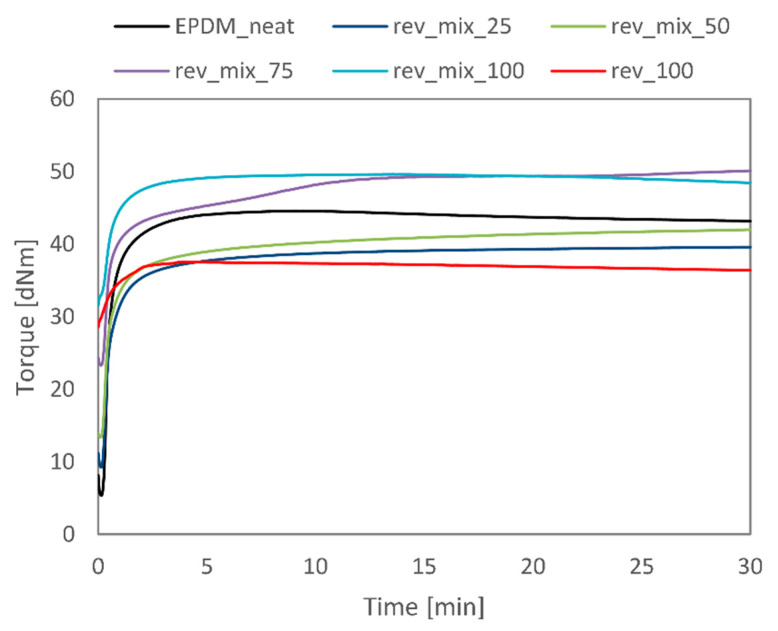
Curing curves for the EPDM revulcanisates.

Based on Figure 2, we chose dev_80-1.33 and dev_80-1.67 for further experiments because they had the highest degree of devulcanisation while retaining low sol content. However, since these two samples had almost identical properties but dev_80-1.33 was prepared with less energy, we eventually opted for it. Figure 3 shows the curing curves of the revulcanisates that were previously introduced in Table 4. The curing curve of the reference virgin rubber (EPDM_neat), as well as those of the samples containing 25 and 50 wt% devulcanisate (rev_mix_25 and rev_mix_50), are characteristic of standard rubber mixtures. There is a clear trend that the higher the devulcanisate content in a mixture, the higher its minimum torque was. The curing curve of rev_100 is similar to those of other devulcanisates in the literature, indicating a low level of curing potential [33,38]. The additional curing agents had a positive effect on the curing properties: the total change in torque for rev_mix_100 was twice as much as that for rev_100. All samples had a long curing plateau, but mixtures containing both virgin rubber and devulcanisate had a marching modulus, which was particularly obvious for rev_mix_75. This can be explained by the slow diffusion of curing agents from one phase to the other. For mixtures containing either devulcanisate only or virgin rubber only, reversion started after 10–15 min.

Furthermore, we presented the most relevant curing parameters in Table 6. Scorch time was between 12 and 16 s for all samples except for rev_100, for which this parameter could not be identified. Optimal cure times increased with increasing devulcanisate content. This phenomenon is not evident from the curing curves, because all curves reached a relative plateau after 50–100 s. However, the shift in cure times can be attributed to the marching behaviour of the cure for samples containing a mixture of virgin and recycled rubbers.

We prepared rubber sheets from each mixture via compression moulding them for their corresponding t_90_. It was possible to cut mechanical test specimens from all sheets, except for rev_100. Even though Figure 3 showed some curing potential for rev_100, the rubber crumbs did not stick to each other, despite the large temperature and pressure. The resulting sheets would disintegrate under their own weight. It can be concluded that the additional curing system is pivotal to achieving 100% recycled rubber via devulcanisation.

**Figure 4:**
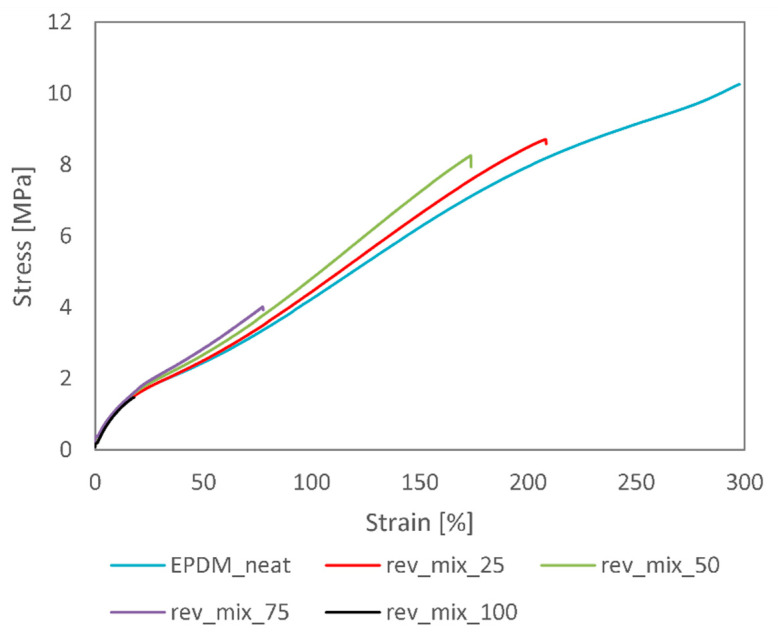
Characteristic stress–strain curves for the EPDM revulcanisates.

**Figure 5:**
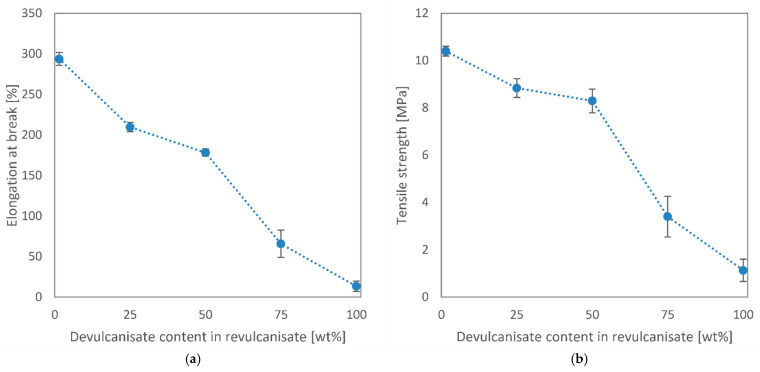
Tensile properties of the revulcanisates plotted against their recycled contents: (**a**) elongation at break vs. devulcanisate content; (**b**) tensile strength vs. devulcanisate content.

Characteristic tensile curves for all revulcanised samples are presented in Figure 4, while the tensile properties of the revulcanisates are further detailed in Figure 5.

As visible in Figure 4, the stress–strain curves overlap at low strains. However, higher recycled contents would shift the curves somewhat higher, indicating an increase in Young’s modulus. It can also be observed that the larger the recycled content, the lower the elongation at break of the samples. Therefore, despite increasing the modulus, the tensile strength of the samples decreased significantly. The diagrams in Figure 5 further emphasise the fact that there was a significant drop in tensile properties between 50 wt% and 75 wt% devulcanisate content. Similar trends were observed in the literature. Yun et al. [21] reported a 35%, 45%, 50%, and 70% decrease in tensile strength and elongation at break for revulcanisates containing 25, 50, 75, and 100 wt% devulcanisate, respectively, compared to virgin rubber. They also observed a slight increase in Young’s modulus. These results were achieved after ultrasonic devulcanisation with a 60% decrease in cross-link density. The relatively small deterioration in tensile properties that we achieved at 50 wt% devulcanisate content is unique.

**Figure 6:**
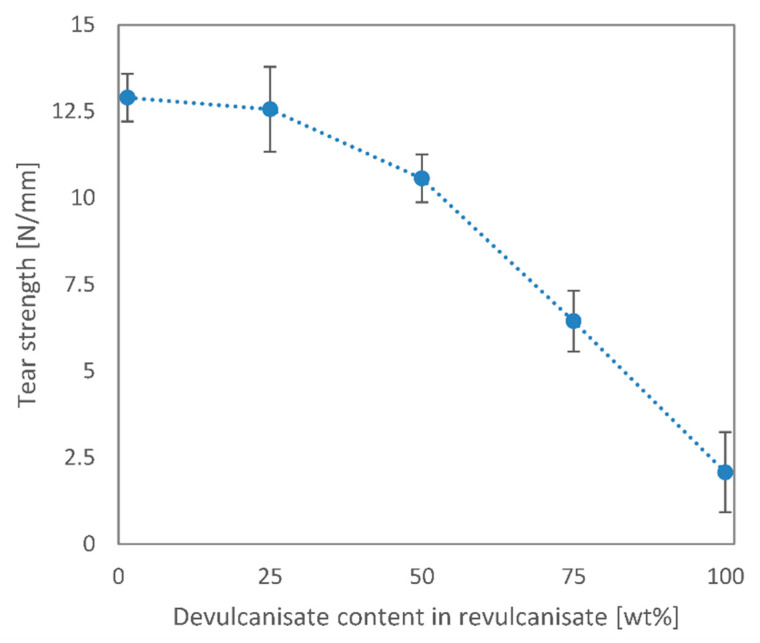
Tear strength of revulcanisates vs. devulcanisate content.

According to Figure 6, tear strength was not affected by low recycled contents as much as tensile properties were. The tear strength decreased by 3% and 18% for 25 wt% and 50 wt% recycled contents, respectively. This phenomenon might have occurred because the devulcanisate crumbs integrated well into the virgin matrix at low recycled contents. However, above 50 wt%, the virgin rubber can no longer surround the crumbs, hence the rapid decrease in mechanical properties. These results further emphasise that the applicability of rev_mix_50 is similar to that of the virgin EPDM rubber, even though this sample contains 50 wt% recycled rubber.

**Figure 7:**
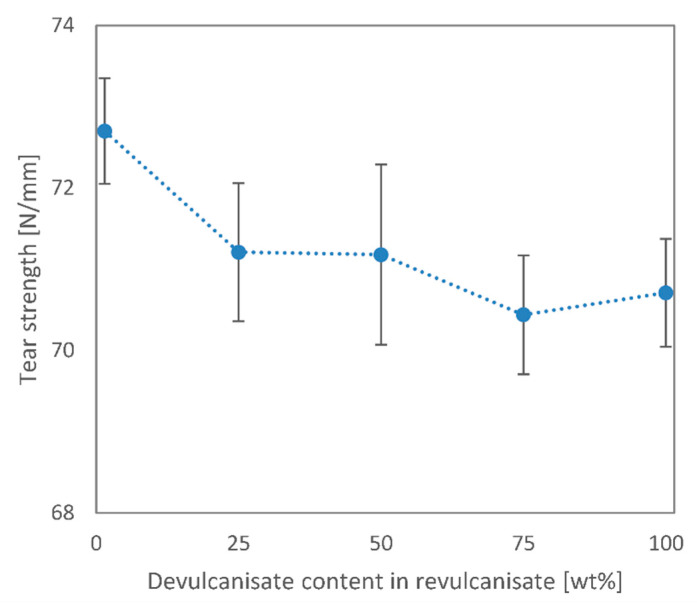
Shore A hardness of EPDM revulcanisates vs. devulcanisate content.

The Shore A hardness of the revulcanisates is presented in Figure 7. All values are relatively close to each other, especially if we consider their standard deviation. There was a minor decrease in hardness as virgin rubber was replaced by recycled rubber. The recycled rubber appears to be slightly less hard than the virgin rubber. The hardness of the samples containing both virgin and recycled rubber does not follow the rule of mixtures, however. The soft devulcanisate phase may be able to absorb the deformation inflicted on the samples, and consequently the change in the Shore A hardness of the samples appears to be stepwise, rather than gradual. This behaviour may be attributed to a good adhesion between the two phases.

**Figure 8:**
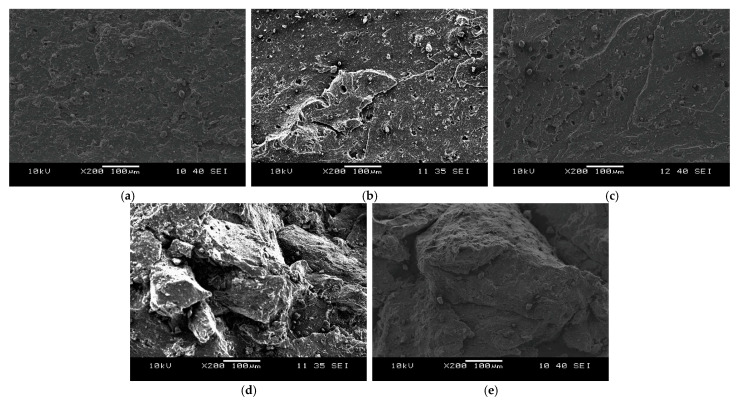
SEM micrographs of the fracture surfaces of the revulcanisates: (**a**) EPDM_neat; (**b**) rev_mix_25; (**c**) rev_mix_50; (**d**) rev_mix_75; (**e**) rev_mix_100.

We took scanning electron micrographs of the fracture surfaces of the tensile test specimens (Figure 8). A relative smoothness of the fracture surface was retained up to 50 wt% devulcanisate content. However, the devulcanisate crumbs did not blend with the virgin rubber at 75 wt% concentration. At 100 wt% concentration, the original rubber crumbs are clearly visible, indicating that they did not merge completely. These morphological findings correlate well with the mechanical properties, in which there was a sharp decline above 50 wt% recycled content.

## 4. Conclusions

We devulcanised a sulphur-cured EPDM rubber of known composition on a two-roll mill at moderate temperatures. We tested three temperature settings (80, 100, and 120 °C) and three friction settings (1.33, 1.67, and 2.00). We achieved a 53% decrease in cross-link density at 80 °C and 1.33 friction, while keeping polymer degradation at a minimum, which is supported by Horikx’s analysis.

We decided that the effectiveness of devulcanisation should be further analysed by evaluating the applicability of the devulcanisate. Hence, we tried revulcanising the devulcanisate on its own. Though the rheometer showed a small increase in modulus during this recuring process, the resulting rubber sheet disintegrated into crumbs. Based on this experience, we added curing agents to the devulcanisate and prepared rubber mixtures containing various amounts of devulcanisate and virgin EPDM rubber.

We performed mechanical and morphological tests on these rubber blends and found that most mechanical and structural properties may be retained up to a 50 wt% devulcanisate content. The elongation at break reduced from around 300% to 180%, while the tensile strength only decreased by 20%. Tear strength and hardness values also barely deteriorated at 50 wt% recycled content. We analysed the tensile test specimens’ fracture surfaces with a scanning electron microscope and found that a relatively smooth fracture surface can be achieved only up to 50 wt% recycled content. At higher devulcanisate contents, the virgin EPDM rubber cannot surround the devulcanised rubber crumbs, which did not stick sufficiently to one another.

Overall, the recycling method presented in this paper is indeed suitable for the recycling of EPDM rubber with a mild reduction in product quality at 25 and 50 wt% recycled contents. The addition of extra curing agents to the devulcanisate is crucial in achieving uniform product quality.

## Figures and Tables

**Table 1:** Composition of the EPDM rubber mixture used for the devulcanisation experiments.

Component	Concentration [phr]
Dutral TER 4047	100
Zinc oxide	4
Zinc stearate	1
UltraLube UL160	3
PEG 4000 ^1^	1
Dolomite B	30
N550 carbon black	45
N772 carbon black	40
DK 350 oil	15
TBTD ^2^	0.8
MBT ^3^	1.5
ZDBC ^4^	0.8
Sulphur	1
Total	243.1

^1^ PEG 4000 stands for polyethylene glycol with an average molecular mass of 4000, ^2^ TBTD stands for tetrabutylthiuram disulphide, ^3^ MBT stands for mercaptobenzothiazole, ^4^ ZDBC stands for zinc dibutyl dithiocarbamate.

**Table 2:** Summary of devulcanisation parameters and devulcanisate nomenclature.

		**Speed of Rolls [rpm]**		
		roll 1: 3	roll 1: 3	roll 1: 3
		roll 2: 4	roll 2: 5	roll 2: 6
		**Friction** [–]		
		1.33	1.67	2.00
**Roll temperature [°C]**	80	dev_80-1.33	dev_80-1.67	dev_80-2.00
	100	dev_100-1.33	dev_100-1.67	dev_100-2.00
	120	dev_120-1.33	dev_120-1.67	dev_120-2.00

**Table 3:** Composition of dev_80-1.33_mix.

Component	Concentration [phr]
dev_80-1.33	243.1
Zinc oxide	4
Zinc stearate	1
TBTD	0.8
MBT	1.5
ZDBC	0.8
Sulphur	1
Total	252.2

**Table 4:** Composition and nomenclature of EPDM revulcanisates.

Sample Name	dev_80-1.33_mix Content [wt%]	Virgin EPDM Content [wt%]	dev_80-1.33 Content [wt%]
EPDM_neat	0	100	0
rev_mix_25	25	75	0
rev_mix_50	50	50	0
rev_mix_75	75	25	0
rev_mix_100	100	0	0
rev_100	0	0	100

**Table 5:** The degree of devulcanisation and sol content of the devulcanisates.

Sample Name	Degree of Devulcanisation [%]	Sol Content [wt%]
dev_80-1.33	53 ± 4.2	5.4 ± 0.1
dev_80-1.67	53 ± 4.4	5.5 ± 0.5
dev_80-2.00	46 ± 5.3	4.0 ± 0.1
dev_100-1.33	40 ± 4.3	2.9 ± 0.5
dev_100-1.67	46 ± 6.0	3.7 ± 1.2
dev_100-2.00	45 ± 5.1	3.9 ± 0.4
dev_120-1.33	50 ± 8.0	4.5 ± 0.6
dev_120-1.67	49 ± 6.5	4.4 ± 0.1
dev_120-2.00	50 ± 4.0	4.2 ± 0.4

**Table 6:** Curing parameters of the revulcanisates.

	EPDM_neat	rev_100	rev_mix_25	rev_mix_50	rev_mix_75	rev_mix_100
Scorch Time [s]	14	–	13	13	15	16
t_90_ [min]	1.72	1.97	2.92	5.45	8.68	2.41
S_min_ [dNm]	5.36	28.55	9.26	13.39	23.30	28.55
S_max_ [dNm]	44.56	37.54	39.58	41.99	50.09	37.54

## Data Availability

Not applicable.
